# 

**Published:** 1890-06-07

**Authors:** 


SATURDAY, JUNE 7th, 1890.

**Imperial Provision for the Sick** - 131
**Words of Consolation.**—Wearisome Days - 132
**Before the Lords' Committee** (By our Special Commissioner). II.—Some Interesting Facts - 133
**The Doctor's Pulpit.**—Golden Pheasants and Clerical Guns 135
**Do the Sick Fear Death?** - 136
**The Military Exhibition** - 137
**Everybody's Page** - 137
**Notes and News** - 138
**Glances at Foreign Asylums** - 139
**Annotations.**—Unsectarian Hospitals—Count Mattei, the Cancer Curer—Merry May at Paddington - 140
**A Year's Work in the Medical Charities of London** - 141
**Scraps and Gleanings** - 144
**Students' Column** - 144

### EXTRA SUPPLEMENT—THE NURSING MIRROR.

**En Passant.**—Zenana Mission at Lucknow—The Oldest Nursing Institution—A Home of Rest for Nurses—A Note from the North—Hypnotism—A Nurse and her Husband—Short Items—Newark News - xxxvii

**The British Nurses' Association and its Solicitor** xxxviii

**The Joys of Johannesberg** - xxxviii

**Nurse Joyce.**—Part II. - xxxix

**Everybody's Opinion.**—Rural Nursing Association—Holiday Homes - xl

**Death in our Ranks** - xl

**Lecture** - xl

**Notes and Queries** - xl

**Vacancies—Appointments** - xl

**Amusements and Relaxation** - xl

